# Human umbilical cord mesenchymal stem cells-derived exosomes exert anti-inflammatory effects on osteoarthritis chondrocytes

**DOI:** 10.18632/aging.205034

**Published:** 2023-09-18

**Authors:** Shichao Wang, Wenyue Jiang, Shuang Lv, Zhicheng Sun, Lihui Si, Jinxin Hu, Yang Yang, Dingbang Qiu, Xiaobin Liu, Siying Zhu, Lunhao Yang, Ling Qi, Guangfan Chi, Guiqing Wang, Pengdong Li, Baojian Liao

**Affiliations:** 1 The Sixth Affiliated Hospital of Guangzhou Medical University, Qingyuan People's Hospital, Qingyuan 511518, Guangdong Province, People's Republic of China; 2 Obstetrics and Gynecology of Sino-Japanese Friendship Hospital of Jilin University, Changchun 130033, Jilin Province, People's Republic of China; 3 The Key Laboratory of Pathobiology, Ministry of Education, Department of Pathology, College of Basic Medical Sciences, Jilin University, Changchun 130021, Jilin Province, People's Republic of China; 4 The Department of Obstetrics and Gynecology, Second Hospital of Jilin University, Changchun 130041, Jilin, People’s Republic of China

**Keywords:** exosomes, mesenchymal stem cells, human umbilical cord, osteoarthritis, chondrocyte

## Abstract

Inflammation of chondrocytes plays a critical role in the occurrence and development of osteoarthritis (OA). Recent evidence indicated exosomes derived from mesenchymal stem cells (MSCs-Exos) exhibit excellent anti-inflammatory ability in many troublesome inflammatory diseases including OA. In the present study, we aimed to explore the role of human umbilical cord-derived MSCs-Exos (hUC-MSCs-Exos) in treating the inflammation of chondrocytes and its related mechanisms. Ultracentrifugation was applied to isolate hUC-MSCs-Exos from the culture supernatant of hUC-MSCs. Two OA-like *in vitro* inflammation models of human articular chondrocytes induced with interleukin 1β (IL-1β) and co-incubation with macrophage utilizing transwell cell culture inserts were both used to evaluate the anti-inflammatory effects of hUC-MSCs-Exos. The mRNA sequencing of chondrocytes after treatment and microRNA (miRNA) sequencing of hUC-MSCs-Exos were detected and analyzed for possible mechanism analysis. The results of the study confirmed that hUC-MSCs-Exos had a reversed effect of IL-1β on chondrocytes in the expression of collagen type II alpha 1 (COL2A1) and matrix metalloproteinase 13 (MMP13). The addition of hUC-MSCs-Exos to M1 macrophages in the upper chamber showed down-regulation of IL-1β and tumor necrosis factor α (*TNF-α*), up-regulation of *IL-10* and arginase1 (*ARG1*), and reversed the gene and protein expression of COL2A1 and MMP13 of the chondrocytes seeded in the lower chamber. The results of this study confirmed the anti-inflammatory effects of hUC-MSCs-Exos in the human articular chondrocytes inflammation model. hUC-MSCs-Exos may be used as a potential cell-free treatment strategy for chondrocyte inflammation in OA.

## INTRODUCTION

Osteoarthritis (OA) is well accepted as a low-grade inflammatory disease that causes pain and dysfunction, seriously endangering the physical and mental health of over 250 million people worldwide [1]. Although the primary etiology of the disease is undetermined, growing evidences indicate that the chondrocyte inflammatory response induced by elevated levels of pro-inflammatory cytokines plays a critical role in the pathogenesis of OA [2, 3]. Anti-inflammatory substances have the potential to be used in the treatment of OA by inhibiting chondrocyte inflammation, as OA pain is believed to be caused by inflammation responsible for OA progression [4]. Nonsteroidal anti-inflammatory drugs (NSAIDs) are widely used in OA therapy for anti-inflammation and pain relief. Nonetheless, these medications solely provide relief from symptoms without impacting the modification or progression of the disease. Therefore, it is imperative to seek novel agents for the anti-inflammation of the chondrocyte in OA.

Recently, increasing focus has focused on the anti-inflammatory ability of mesenchymal stem cells (MSCs) [5–7]. For the treatment of OA, local injection of MSCs is an effective intervention. Nevertheless, the cells require strict storage and transportation conditions. In addition, the differentiation for non-therapeutic purposes, and risk of disease transmission, and possible immune rejection of MSCs hinder the application [8]. Exosomes are the extracellular vesicle secreted by cells to the outside of the cell. Recent evidence suggests that MSCs-derived exosomes (MSCs-Exos) possess similar biological functions to MSCs [9]. While compared with MSCs, it is more stable under different pathophysiological conditions and hardly causes immune rejection in the body. These unique advantages make MSCs-Exos a hotter spot than MSCs.

In recent years, bone marrow-derived MSCs-Exos and synovial-derived MSCs-Exos have been reported to inhibit articular chondrocytes inflammation [8, 10–13]. The underlying mechanisms were reported via p38, ERK, Akt, HDAC3, and NF-κB pathways. The microRNAs (miRNAs) carried by exosomes are implicated in anti-inflammatory biological processes. While there was no research on the human umbilical cord-derived MSCs-Exos (hUC-MSCs-Exos) for the anti-inflammatory effects of chondrocytes. hUC-MSCs are derived from discarded umbilical cord tissue, with the advantages of easy obtaining, which were considered important MSCs-Exos sources.

In this study, we aimed to explore the role of hUC-MSCs-Exos in treating the inflammation of chondrocytes and its related mechanisms. Here, we used interleukin 1β (IL-1β) and macrophage co-incubation to induce OA-like inflammation in human chondrocytes. Then the potential anti-inflammatory effects of hUC-MSCs-Exos on human chondrocytes were investigated. Furthermore, through mRNA sequencing of chondrocyte after treatment and miRNAs sequencing of hUC-MSCs-Exos, underlying mechanism have been explored.

## MATERIALS AND METHODS

### Culture and identification of hUC-MSCs

### Isolation and culture of hUC-MSCs

The Ethics Committee of the Second Hospital of Jilin University and the Ethics Committee of the Qingyuan People's Hospital granted approval for human umbilical cord collection and all experimental procedures (approval no.: 2019-142, IRB-2023-017).

After obtaining approval and patient consent, umbilical cord was obtained from a healthy newborn at term. For the detailed method of hUC-MSCs isolation, refer to the previous literature [14]. Umbilical cord tissues were rinsed 3 times with phosphate buffer (PBS; ThermoFisher) containing 5% Penicillin/Streptomycin (P/S; ThermoFisher). Umbilical vein and umbilical artery were removed, and Wharton’s jelly tissue was retained. Wharton's jelly tissue was cut into small pieces (about 0.5×0.5mm), attached to petri dishes, and dropped into medium containing 10 ng/mL basic fibroblast growth factor (bFGF; PeproTech), 1% P/S, 10% fetal bovine serum (FBS; ThermoFisher) and 89% Dulbecco's modified eagle medium nutrient mixture F-12; (DMEM/F12; ThermoFisher) for culture. When the confluence of the cells reached approximately 90%, the cells were rinsed with PBS and then digested with 0.25% EDTA-trypsin (ThermoFisher). The cells were centrifuged at 300× *g* and subculture. The fifth passage of cells was used for identification and exosome collection experiments.

### Immunofluorescence staining

An immunofluorescence staining assay was conducted to identify surface markers. The primary antibodies mainly include: anti-CD31 (1:100; MA1-26196, ThermoFisher), anti-CD44 (1:1000; MAB7045, R&D Systems, UK), anti-CD45 (1:100; 16-0451-85, ThermoFisher), anti-CD73 (1:100; 41-0200, ThermoFisher), anti-CD90 (1:100; PA5-82115, ThermoFisher) and anti-CD105 (1:100; MA5-17041, ThermoFisher). hUC-MSCs were incubated overnight with primary antibodies and, after rinsing, incubated with secondary antibodies (Alexa Fluor 488; 1:1000; CST). Hoechst 33342 (10 μg/mL; ThermoFisher) is utilized to stain the nucleus of hUC-MSCs. Images of stained hUC-MSCs were acquired by fluorescence microscope. The negative control consisted of cells stained only with the secondary antibody.

### Differentiation ability

Multilineage differentiation potential of hUC-MSCs was examined by chondrogenic, adipogenic and osteogenic differentiation assays. For chondrogenic differentiation, 20 μL suspension drop the fifth passage of hUC-MSCs (8 × 10^6 cells/mL) were cultured into chondrospheres Subsequently, these spheres were cultured in chondrogenic induction medium consisting of high glucose-Dulbecco's Modified Eagle Medium (HG-DMEM; ThermoFisher) supplemented with 10% FBS, 50 nM of ascorbate-2-phosphate (Sigma-Aldrich), 10 ng/mL transforming growth factor-beta 1 (PeproTech, USA) and 6.25 μg/mL insulin. The induction medium was changed at 3-day intervals. After three weeks of chondrogenic induction and histopathological manipulation the tissue sections of cartilage spheres were stained with toluidine blue (Dingguo, China) according to the manufacturer's protocol. When the fusion degree of the fifth passage of hUC-MSCs was about 80%, adipogenic induction solution, which contained HG-DMEM supplemented with 10% FBS, 1% P/S, 0.5 mM isobutyl-methylxanthine (Sigma-Aldrich), 10 μM insulin (Sigma-Aldrich), 200 μM indomethacin (Sigma-Aldrich) and 1 μM dexamethasone (Sigma-Aldrich) was added. The induction medium was changed at 3-day intervals. After continuous culture for 2 weeks, the lipid inclusion vacuoles were detected using oil red O (Sigma-Aldrich) according to the previous methods of our research group [15]. When the fusion degree of the fifth passage of hUC-MSCs was about 60%, osteogenic induction solution, which contained HG-DMEM supplemented with 10% FBS, 1% P/S, 50 μM ascorbate-2-phosphate (Sigma-Aldrich), 0.1 μM dexamethasone and 10 nM β-glycerophosphate (Sigma-Aldrich) was added. The induction medium was changed at 3-day intervals. After continuous culture for 3 weeks, the calcium salt was stained with alizarin red S (Sigma-Aldrich) according to the previous methods of our research group [15]. Images were recorded using an inverted fluorescence microscope.

### Isolation and identification of hUC-MSCs-Exos

### Isolation of hUC-MSCs-Exos

Ultracentrifugation was used to obtain hUC-MSCs-Exos [16]. When hUC-MSCs had grown to approximately 50% confluence, they were rinsed once with PBS and replaced with exosomes-free medium for 48 hours (containing 89% DMEM/F-12, 10 ng/mL bFGF, 10% FBS without exosomes (SBI, USA) and 1% P/S). The hUC-MSCs culture supernatant was centrifuged at 300 ×*g* for 10min at 4° C in order to remove the cells. The hUC-MSCs culture supernatant from which the cells were removed was centrifuged for 10min at 2,000×*g* in order to remove debris of cells. Subsequently, supernatants collected from the above experiments were centrifuged at 10,000×*g* for 30min at 4° C in order to remove organelles. After serial centrifugations, the supernatant of hUC-MSCs culture medium was centrifuged by ultracentrifugation at 120,000 ×*g* for 90 min at 4° C using a SW32Ti rotor (Beckman Coulter, USA) to obtain hUC-MSCs-Exos. The hUC-MSCs-Exos were rinsed once with PBS and centrifuged by ultracentrifugation to remove the mixed proteins. The hUC-MSCs-Exos were dissolved in 400ul PBS, packaged separately, and stored at -80° C in a refrigerator for future use.

### Identification of hUC-MSCs-Exos

To identify hUC-MSCs-Exos, a series of experiments were conducted. The number of hUC-MSCs-Exos was detected utilizing ExoELISA-ULTRA (CD81 detection; System Biosciences, USA) according to the instructions. The size distribution of hUC-MSCs-Exos was detected utilizing ZETASIZER Nano series-Nano-ZS (Malvern Instruments, UK). The data was analyzed utilizing zetasizer software (Malvern Instruments). Additionally, the morphology of the hUC-MSCs-Exos was observed by transmission electron microscopy (TEM). Briefly, the hUC-MSCs-Exos were dropped-added to the copper mesh, fixed by adding 2% uranyl acetate (Sigma-Aldrich) and allowed to air dry at room temperature. The images of hUC-MSCs-Exos were acquired through TECNAI 12 TEM (FEI, USA). The markers of exosome were assessed using western blotting, with antibodies against CD81 (1:1000; ab79559, Abcam), CD9 (1:1000; 13403S, Cell Signaling), TSG101(1:1000; AF8262, Beyotime) and CD63 (1:1000; ab271286, Abcam, UK). Expression ratio of exosome markers were quantified by flow cytometry (BD Bioscienes, USA). hUC-MSCs-Exos were incubated with CD81-antibody-PE (1:5; BD) and CD63-antibody-FITC (1:5; BD) for 2 h on ice. hUC-MSCs-Exos without treatment were used as the negative control.

### Anti-inflammatory effects of hUC-MSCs-Exos on human chondrocytes

### The effect of hUC-MSCs-Exos on IL-1β-induced human chondrocyte

Human knee chondrocytes (PromoCell, Germany) were cultured using chondrocyte growth medium (PromoCell, Germany), and the third passage chondrocytes were used for subsequent experiments. The method used to replicate the cartilage inflammation model was previously reported in the literature [17]. After 24 hours of cell seeding, the chondrocyte growth medium was added with IL-1β (10 ng/mL; PeproTech, USA). The chondrocyte growth medium containing hUC-MSCs-Exos (4 × 10^7 particles/mL) or without were changed. After 48 hours, Chondrocytes were harvested and examined accordingly. Normal cultured chondrocytes were used as control.

### hUC-MSCs-Exos co-culture with macrophages and chondrocytes in transwell systems

Chondrocytes were co-cultured with macrophages using 0.4 μm permeable cell culture chambers (Corning, USA) Briefly, THP-1 cells were seeded on the upper chambers of the permeable cell culture chambers at a density of 5 × 10^5 cells/chamber in 12-well with 25 ng/mL PMA for 24 h, followed by polarization to M1 macrophages with 100 ng/mL LPS for 24 h. Meanwhile, chondrocytes were seeded in the lower chamber of the permeable cell culture chambers at a density of 5 × 10^4 cells/chambers for 24 h. For further incubation with hUC-MSCs-Exos, macrophages chambers were washed with PBS, moved into chondrocytes chamber, added fresh hUC-MSCs-Exos in the upper chamber for 48 h. The co-culture system with LPS and hUC-MSCs-Exos were supplemented as the experimental group, the one without hUC-MSCs-Exos was used as the control group, and the one without LPS and hUC-MSCs-Exos were used as the blank control group.

### RT-qPCR

Total RNA was extracted applying trizol (ThermoFisher, USA) according to the manufacturer's instructions. cDNA was synthesized utilizing Titan One Tube RT-PCR kit (Roche, Switzerland) and according the manufacturer’s guidelines. qPCR was conducted using FastStart Universal SYBR Green Master (Roche) according to the manufacturer's instructions on CFX Connect Real-Time System (Bio-Rad, Germany). The expression abundance of the detected mRNA was calculated using the 2^(-ΔΔCT) method. RT-qPCR primers targeting *GAPDH* (NM_001256799.2), *IL-1β* (NM_000576.2), *ARG1*(NM_000045.2), *IL-10* (NM_000572.2), *COL2A1* (NM_033150.2), *MMP1*3 (NM_002427.3) and *TNF-α* (NM_001286813.1) were procured from Guangzhou GeneCopoeia Co., Ltd (China).

### Immunofluorescence staining

Chondrocytes were incubated with antibodies against MMP13 (1:1000; MAB511, R&D Systems) and COL2A1 (1:100; MA5-12781, ThermoFisher), followed by corresponding secondary antibody (Alexa Fluor 488, 1:1000). Hoechst 33342 is utilized to stain the nucleus of chondrocytes. Images of stained chondrocytes were acquired by fluorescence microscope.

### TUNEL staining

Chondrocytes were washed with PBS, fixed with 4% paraformaldehyde for 30min, and permeabilized with 0.1% TritonX-100 for 15min. And then, chondrocytes were stained with *In Situ* Cell Death Detection Kit (Roche) according to the instructions. Chondrocytes were visualized under an inverted fluorescence microscope after counterstaining with hoechst 33342 (10 μg/mL).

### Exos small RNA sequencing and data analysis

### Exosomal miRNA sequencing and bioinformatics analysis of miRNA target genes

Exosomal miRNA sequencing was performed by Ribobio Co. Ltd (China). Exosomes derived from three volunteers' hUC-MSCs were employed.

The reads that remained after filtering were mapped to the miRbase database (v22) to quantify their expression against known miRNAs. The read count for each miRNA was obtained by performing comparison and correction. The expression abundance of hUC-MSCs exosomes in the top 50 miRNAs was screened. Target gene of the top 50 miRNAs prediction and bioinformatics analysis were performed. The most significant terms/pathways for the three primary categories (molecular functions, biological processes and cell components) in the Gene Ontology (GO) database, as well as the Kyoto Encyclopedia of Genes and Genomes (KEGG) pathways, were displayed.

### Chondrocyte RNA sequencing and data analysis

After treatment by IL-1β with and without hUC-MSCs-Exos for 48 h, the total RNA of chondrocytes was extracted applying Trizol. mRNA sequencing was performed by Ribobio Co. Ltd (China). The genes up-regulated in IL-1β treated chondrocyte, while genes down-regulated after being treated with hUC-MSCs-Exos and relative to OA target genes (GeneCards database, https://www.genecards.org/), were take intersection by Venn diagrams and used to further data analysis. KEGG and GO analyses were carried out on the intersection mRNAs.

### Statistical analysis

All results were repeated at least three times. Data was presented as the mean ± standard deviation. GraphPad Prism 7.0 software was used to analyze the data. Independent-sample t-test was used to compare two different groups, while ANOVA was employed for statistical analysis of multiple groups of data. *P* < 0.05 was considered statistically significant.

### Availability of data and material

The datasets generated during the current study are available.

### Consent for publication

Consent for publication was obtained from all participants of this study.

## RESULTS

### Isolation and identification of hUC-MSCs

**Figure 1:**
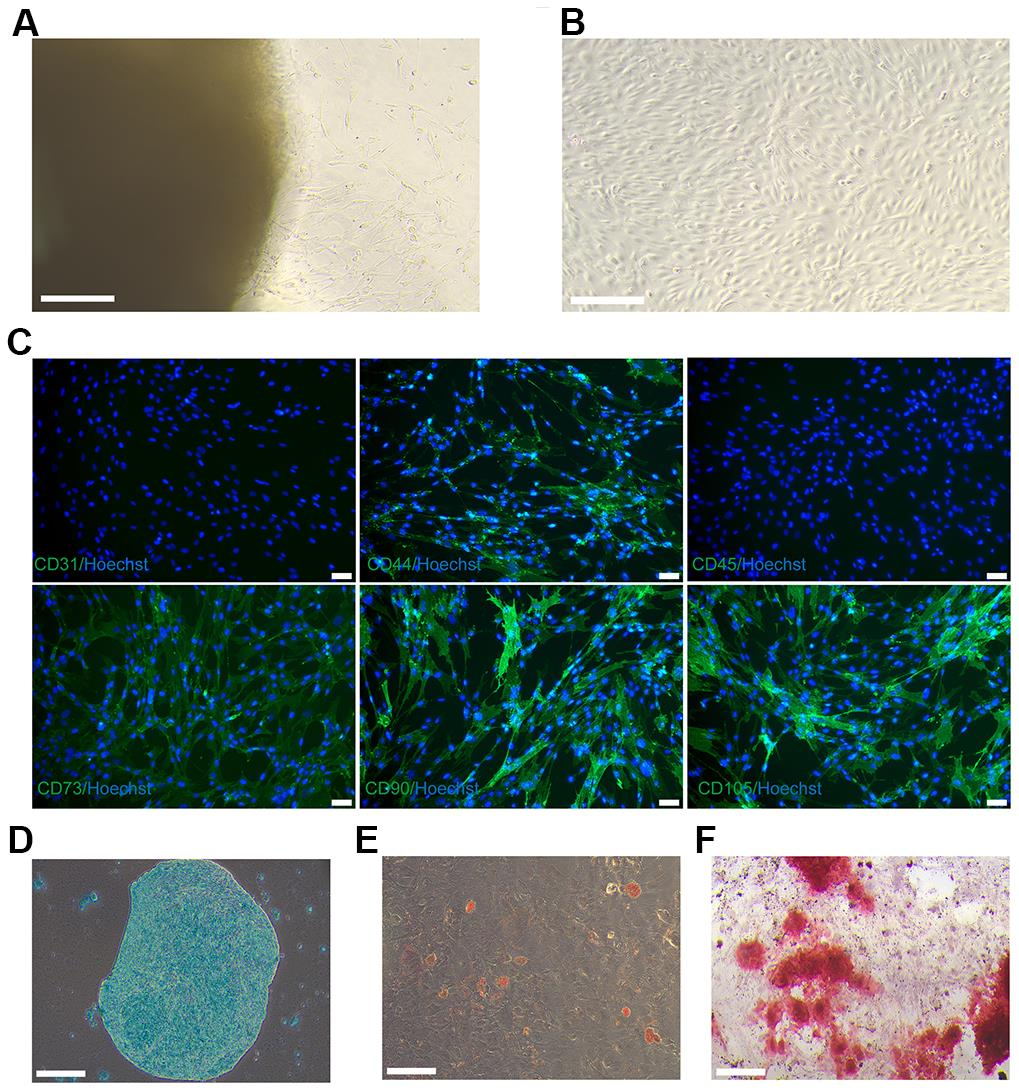
**Culture and identification of hUC-MSCs.** (**A**) On day 12, hUC-MSCs migrated from the Wharton’s jelly tissue. Scale bar=200 μm. (**B**) Images of long-scaled hUC-MSCs cultured for the fifth passage. Scale bar=200 μm. (**C**) Immunofluorescence staining of hUC-MSCs with surface markers CD44, CD73, CD90, and CD105 (green fluorescence). Hoechst 33342 (blue) was utilized to stain the nucleus of hUC-MSCs. Scale bar=50 μm. (**D**) Toluidine blue staining was used to detect the ability of hUC-MSCs to differentiate into chondrocytes. Scale bar=200 μm. (**E**) Oil red O staining was used to detect the ability of hUC-MSCs to differentiate into adipocytes. Scale bar=200 μm. (**F**) Alizarin red S staining was utilised to test the capacity of hUC-MSCs to differentiate into osteoblasts. Scale bar=200 μm.

Cells emerged from the Wharton’s jelly tissue between days 7 and 14 and propagated in cell-culture dish, as revealed in Figure 1A. Cells derived from Wharton’s jelly tissue displayed a spindle-shaped morphology, as depicted in Figure 1B. The fifth passage cells were characterized and utilized in downstream experiments. The cells derived from Wharton’s jelly tissue that expressed the MSCs markers CD90, CD44, CD105 and CD73, but did not express vascular endothelial marker CD31 and leukocyte marker CD45. As revealed in Figure 1C–1F, the cells possessed multipotent differentiation potentials towards chondrocytes, adipocytes, and osteoblasts. Therefore, the cells derived from Wharton’s jelly tissue were considered as hUC-MSCs.

### Identification of hUC-MSCs-Exos

**Figure 2:**
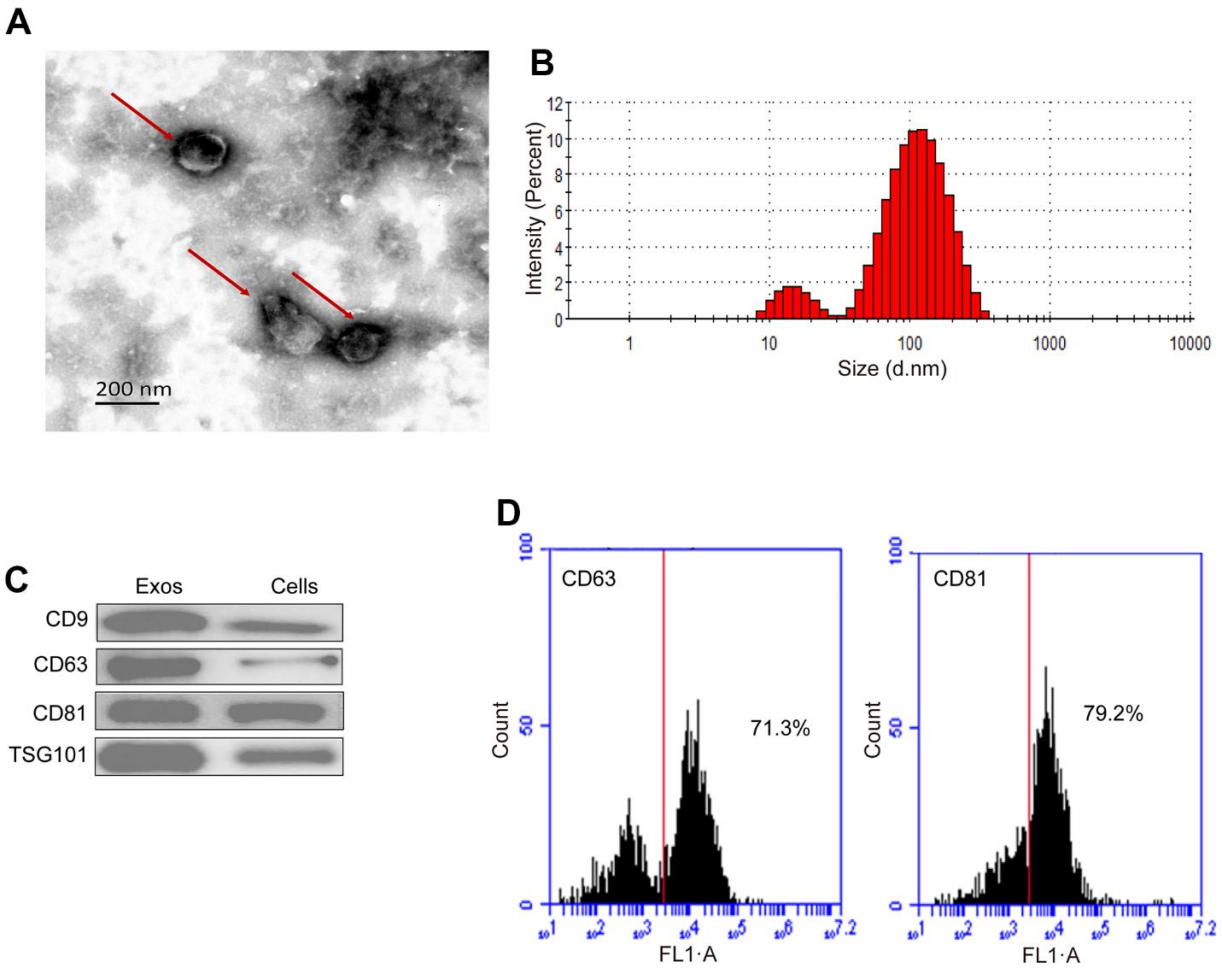
**Characterization of hUC-MSCs-Exos.** (**A**) Representative TEM images of hUC-MSCs-Exos. Scale bar=200 nm. (**B**) The particle size distribution of hUC-MSCs-Exos was detected by ZETASIZER Nano series-Nano-ZS. (**C**) Western blot detected the expression of exosomal markers (CD9, CD63, CD81 and TSG101). (**D**) Flow cytometry detected the proportion of expression of exosomal markers CD63 (71.3%) and CD81 (79.2%).

The oval vesicle of 30-200 nm diameter with a typical membrane structure of exosomes from hUC-MSCs was revealed by TEM, as depicted in Figure 2A. The results of nanoparticle showed that 82.5% of the hUC-MSCs-Exos had distribution rang 30-200 nm, as shown in Figure 2B. The western blotting results showed that the hUC-MSCs-Exos highly expressed the markers of exosome CD9, CD63, CD81 and TSG101 (Figure 2C). Flow cytometry confirmed some markers of exosome CD63 (71.3%) and CD81 (79.2%) (Figure 2D). Based on the aforementioned findings, it can be concluded that the isolation of hUC-MSCs-Exos was successful.

### hUC-MSCs-Exos reverse IL-1β-induced chondrocyte inflammatory response

**Figure 3:**
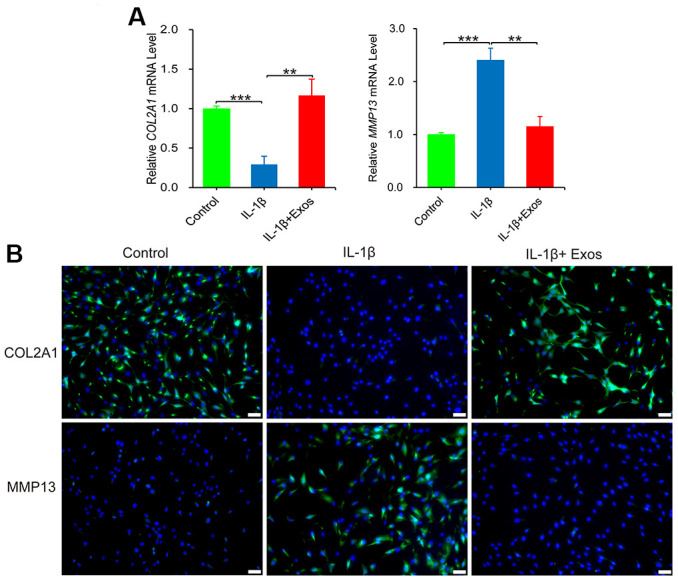
**The anti-inflammatory effect of hUC-MSCs-Exos was evaluated chondrocytes inflammation model.** (**A**) The expressions of *COL2A1* and *MMP13* mRNA of chondrocytes were assayed by qRT-PCR. This experiment was repeated three times. **P < 0.01, ***P < 0.001. (**B**) Immunofluorescence staining was used to detect the protein expression of COL2A1 and MMP13 (green) in chondrocytes. Hoechst 33342 (blue) is utilized to stain the nucleus of chondrocytes. Scale bar=50 μm.

The inflammatory factor IL-1β has a constructive role in the disease progression of OA. IL-1β can induce chondrocytes to produce excess of matrix metalloproteinases (MMP), which causes degradation of extracellular matrix components of chondrocytes, including COL2A1 and aggrecan. *In vitro*, IL-1β is commonly employed to induce inflammation in chondrocytes of OA [18]. Therefore, agents targeting the IL-1β-induced chondrocyte inflammatory response can potentially attenuate the progression of OA [19]. As revealed in Figure 3A, IL-1β downregulated the gene expression of *COL2A1* and upregulated that of *MMP13*, successfully establishing an IL-1β-induced chondrocyte inflammation model. Notably, supplementation with hUC-MSCs-Exos in chondrocyte inflammation model reversed the protein expression of MMP13 and COL2A1, consistent with results of RT-qPCR (Figure 3B). To conclude, hUC-MSCs-Exos demonstrated the ability to alleviate the inflammation of chondrocytes provoked by IL-1β *in vitro* model.

### hUC-MSCs-Exos inhibit M1 macrophage damage to chondrocytes

**Figure 4:**
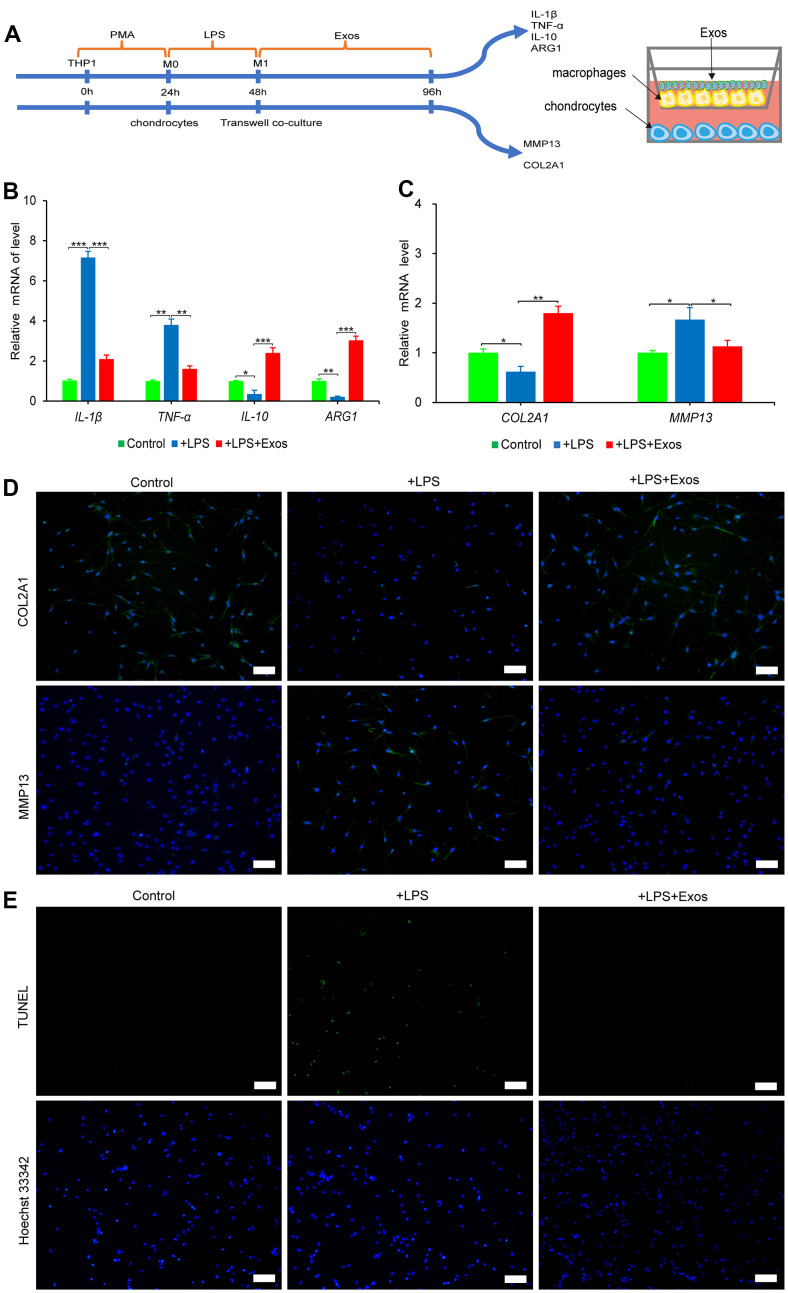
**The anti-inflammatory effect of hUC-MSCs-Exos was evaluated by co-culture system of macrophages and chondrocytes.** (**A**) Graphical abstract of co-culture system of chondrocytes and macrophages. (**B**) The expressions of *IL-1β*, *TNF-α*, *IL-10* and *ARG1* mRNA of macrophages were assayed by qRT-PCR. This experiment was repeated three times. *P < 0.05, **P < 0.01, ***P < 0.001. (**C**) The expressions of *COL2A1 and MMP13* mRNA of chondrocytes were assayed by qRT-PCR. This experiment was repeated three times. *P < 0.05, **P < 0.01. (**D**) Immunofluorescence staining was used to detect the protein expression of COL2A1 and MMP13 (green) in chondrocytes. Hoechst 33342 (blue) is utilized to stain the nucleus of chondrocytes. Scale bar=100 μm. (**E**) TUNEL (green) staining of chondrocytes. Hoechst 33342 (blue) is utilized to stain the nucleus of chondrocytes. Scale bar=100 μm.

Activation and polarization of synovial macrophages to M1 phenotypes play a major role in OA progression [20]. M1 macrophages is activated by LPS, which secretes high levels of pro-inflammatory cytokines, such as IL-1β, TNF-α and low levels of anti-inflammatory factors of ARG1 and IL-10, that lead to inflammation of the chondrocytes [21]. In order to investigate whether hUC-MSCs-Exos inhibit M1 macrophage damage to chondrocytes. A co-culture system of M1 macrophages and chondrocytes was established as shown in Figure 4A. The addition of hUC-MSCs-Exos and LPS to macrophages in the upper chamber resulted in down-regulation of the expression of *TNF-α* and *IL-1β* genes and up-regulation of *ARG1* and *IL-10* genes compared to macrophages with LPS alone (Figure 4B). Chondrocytes co-cultured with macrophages added with hUC-MSCs-Exos and LPS showed upregulated expression of the chondrocyte marker COL2A1 gene and protein and downregulated expression of the chondrocyte catabolic enzyme marker MMP13 gene and protein compared to chondrocytes co-cultured with M1 macrophages (Figure 4C, 4D). M1 macrophages were able to induce apoptosis in chondrocytes. However, when hUC-MSCs-Exos were added, the number of apoptotic chondrocytes was significantly reduced compared to chondrocytes co-cultured with M1 macrophages (Figure 4E). The results demonstrated that hUC-MSCs-Exos had a protective effect against M1 macrophage-induced chondrocyte damage and apoptosis.

### hUC-MSCs-Exos miRNA sequencing and bioinformatic analysis

**Figure 5:**
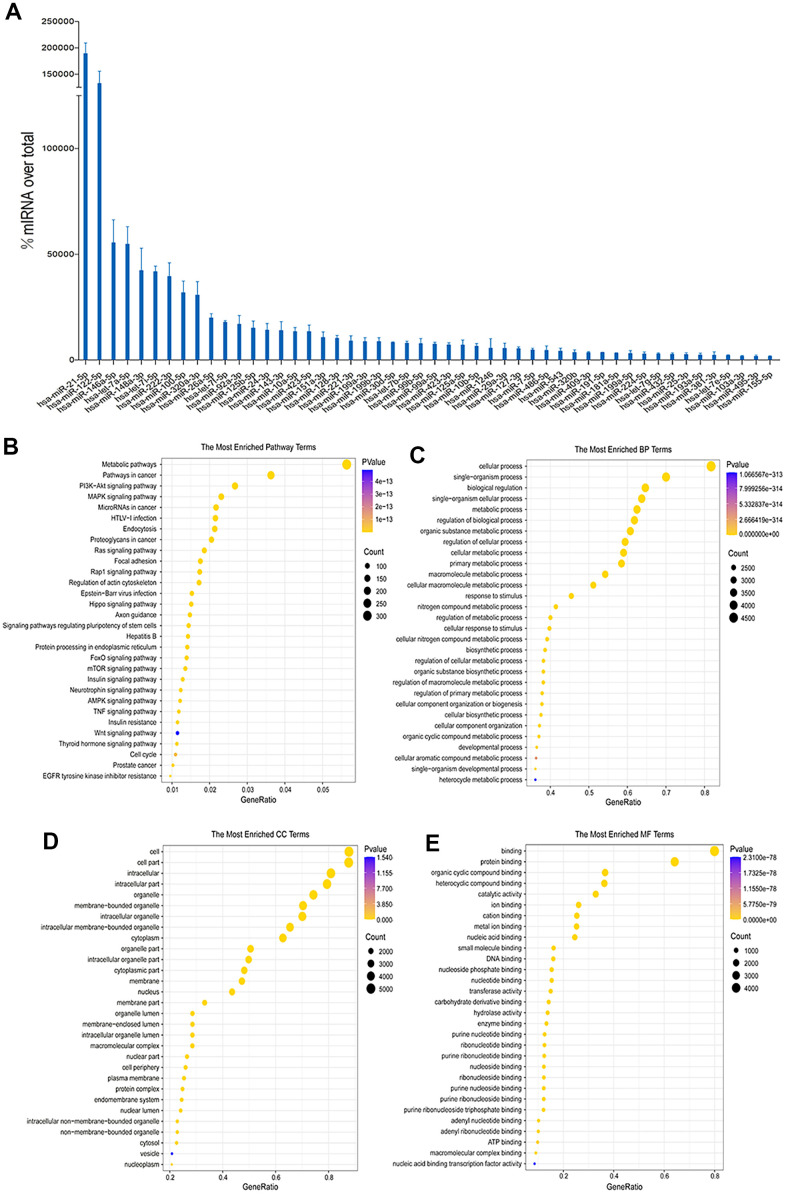
**Sequencing and bioinformatics analysis of miRNAs in hUC-MSCs-Exos.** (**A**) Top 50 highly expressed hUC-MSCs-Exos miRNAs. (**B**–**E**) KEGG and GO (GO molecular function, GO biological process and GO cellular component) analysis of genes regulated by the top 50 highly expressed miRNAs of hUC-MSCs-Exos. The color of bars indicates *p* values, and *p*< 0.05 was considered significant.

Exosomes are rich in a variety of miRNAs, which regulate the biological behavior of cells after binding to target genes. The top 50 high-abundance miRNAs in hUC-MSCs-Exos were screened according to the total read count and presented in Figure 5A. Previous studies have shown there was a high abundance of miRNAs of hUC-MSCs-Exos, such as miRNA-21-5p and miR-146a-3p which play important roles in regulating inflammation of chondrocytes [22, 23]. In chondrocytes, up-regulation of miRNA-21-5p can increase the expression of COL2A1, decrease the expression of MMP13 and ADAMTS5, and regulate the ECM balance of chondrocytes. miRNA-21-5p may be a key miRNA in OA as it promotes hyaline cartilage production [22]. miRNA-146a plays a key role in inflammatory diseases [23]. Furthermore, miRNA-146a induces downregulation of M1 markers and upregulation of M2 markers in macrophages, regulating macrophage polarization [24]. In addition, functional enrichment analysis was able to predict the dominant pathway controlled by miRNA of hUC-MSCs-Exos [23]. The MAPK signaling pathway, metabolic pathway, PI3K-Akt signaling pathway and cancer pathway were the primary KEGG pathway enrichments (Figure 5B). GO analysis depicted that the biological process component was dominated by “single-organism process” and “cellular process”, while the cellular component was characterized by “cell part” and “intracellular.” Additionally, the molecular function component was enriched in “protein binding” and “organic cyclic compound binding,” as demonstrated in Figure 5C–5E. In summary, these conclusions obtained by bioinformatics analysis indicate a possible contribution of miRNAs of hUC-MSCs-Exos in regulating inflammation of chondrocytes.

### Chondrocyte mRNA sequencing and bioinformatic analysis

**Figure 6:**
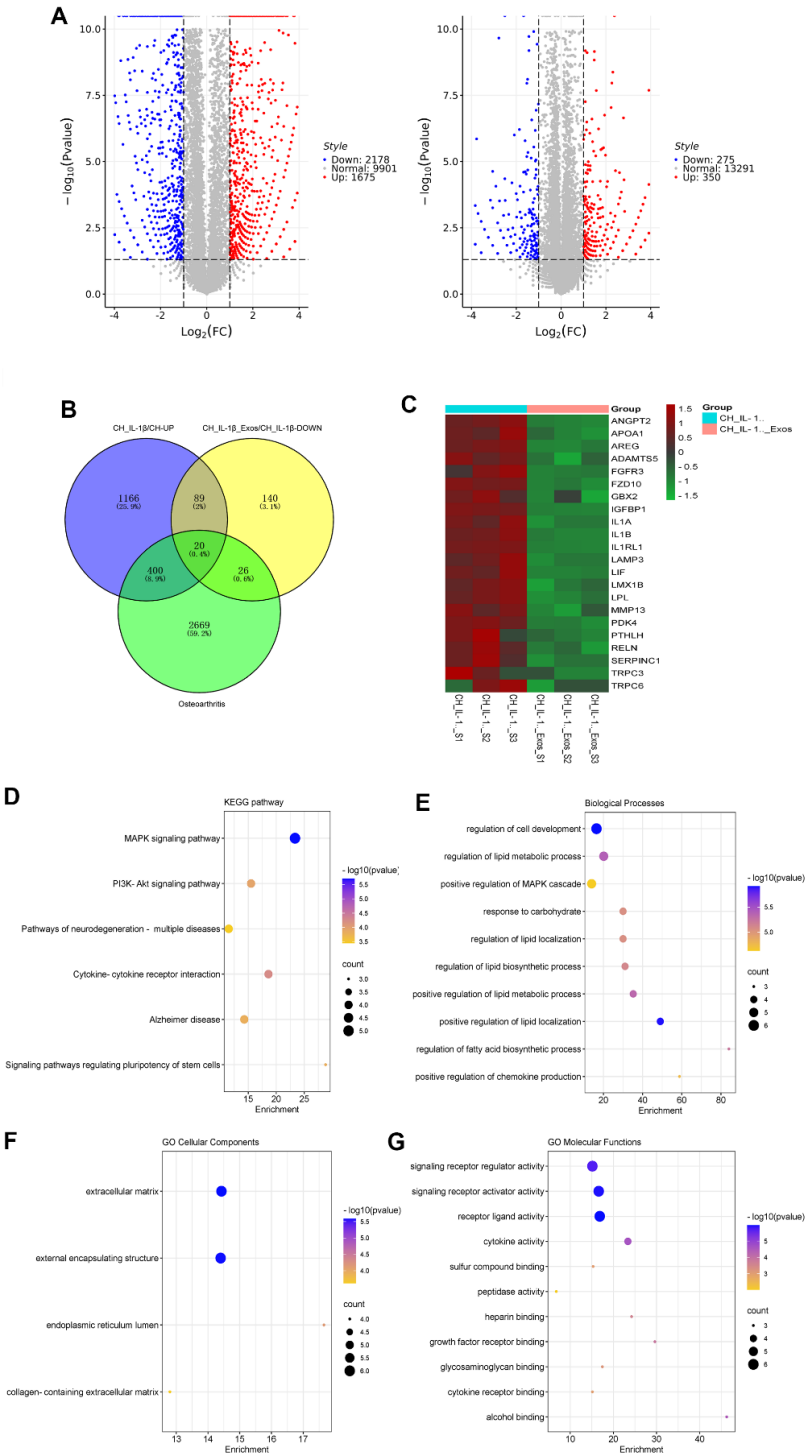
**Sequencing and bioinformatic analysis of mRNA in chondrocytes.** (**A**) Volcano plot of genes differentially expressed by sequencing of chondrocytes from different groups. (**B**) Venn diagram of 1675 genes up-regulated in chondrocytes treated with IL-1β relative to normal chondrocyte, 275 genes downregulated in chondrocytes treated with IL-1β and hUC-MSCs-Exos relative to chondrocytes treated with IL-1β, and 3095 genes related to osteoarthritis in GeneCards database. (**C**) Heatmap of the screened 20 differentially expressed genes. (**D**–**G**) KEGG and GO (GO biological process, GO cellular component and GO molecular function) analysis of the screened 20 differentially expressed genes. The color of bars indicates *p* values, and *p*< 0.05 was considered significant.

As sequencing results showed, there were 1675 genes up-regulated in IL-1β-induced chondrocytes compared to normal chondrocytes, there were 275 genes down-regulated in hUC-MSCs-Exos-treated chondrocyte by induced IL-1β compared to IL-1β-induced chondrocytes (Figure 6A). The osteoarthritis-related genes in the GeneCards database were intersected with the up- and down-regulated genes in the sequencing results, and 22 genes were finally screened (Figure 6B, 6C). KEGG pathway enrichment analysis was performed, and a total of 6 pathways were obtained (Figure 6D). Among them, MAPK and PI3K-Akt is an important signaling pathway for hUC-MSCs-Exos to exert anti-inflammatory effects in OA. GO analysis (Figure 6E–6G) showed that “regulation of cell development process” and “regulation of lipid metabolic process” in the biological process component, “extracellular matrix” and “external encapsulating structure” in the cellular component, “signaling receptor activator activity” and “signaling receptor regulator activity” in the molecular function component had the high significance.

## DISCUSSION

Many inflammatory processes are implicated in the degenerative disease known as OA. Elevated inflammatory mediators could lead to the damage of articular tissues in OA. Therefore, targeting the pathways of inflammation may represent a novel therapeutic strategy for OA. Recently, an increasing amount of research has been directed towards exploring the anti-inflammatory potential of MSCs-Exos, such as the MSCs-Exos derived from bone marrow and synovial have been reported to inhibit articular chondrocytes inflammation [25]. Compared to other MSCs, the hUC-MSCs was easy to obtain with high yield and non-invasive, which is considered an ideal source for exosomes. Recent evidences suggest that hUC-MSCs-Exos have the capacity of promoting osteochondral regeneration [1], inhibit apoptosis and ROS production in chondrocytes [26]. In this study, it has been confirmed that hUC-MSCs-Exos have the effect of resisting chondrocyte inflammation which induced by IL-1β or M1 macrophage. hUC-MSCs-Exos could increased the expression of COL2A1 and decreased MMP13 expression, exert anti-inflammatory effects on chondrocytes. All this together have proved hUC-MSCs-Exos to be a novel agent for treatment of OA.

Macrophages act as immune cells, it plays very important role in the damage of OA articular cartilage cells, and both the inflammatory and destructive responses of OA [27]. Macrophages in OA produce inflammatory factors such as MMP and tissue inhibitors of metalloproteinases, which have destructive effects on articular cartilage [28]. This effect not only involved apoptosis and senescence, but also decreased the synthesis of key ECM components such as COL2A1 in chondrocytes. Additionally, inflammatory factors promote the synthesis and release of proteolytic enzymes in chondrocytes, including MMP and ADAMTS. TNF-α and IL-1β play key role in the pathogenesis of OA. In addition to promoting the inflammatory response in OA, TNF-α and IL-1β can promote osteoclast formation and bone resorption, which in turn stimulates osteoblasts to secrete MMP and cytokines that are detrimental to bone and adjacent cartilage [29].

Some studies have shown that M1 macrophages of synovial may directly lead to chondrocytes damage through production of MMP, causing the OA [30]. Macrophages generally have two phenotypes, a pro-inflammatory M1 type and an anti-inflammatory M2 type, and the ratio of these two phenotypes changes as the disease progresses [31]. Inflammatory factors produced by M1 macrophages and their proteolytic enzymes overexpressed by chondrocytes are the key causes of OA. The interaction of chondrocytes and macrophages displays another mechanism for pathogenesis of OA [32]. M1 macrophages secrete a lot of inflammatory factors such as IL-1β, TNF-α, etc., which promote chondrocytes to produce excess MMP and aggrecanase, and influence the microenvironment of chondrocytes, resulting in ECM components, including COL2A1 and proteoglycan degradation [28].

In the miRNA sequencing results of hUC-MSCs-Exos, in addition to miRNA-21-5p and miRNA-146a, there are miR-26a-5p, miR-100-5p, and let-7a among the highly expressed miRNAs, which play important roles in maintaining cartilage homeostasis and regulating inflammation. High-throughput sequencing in this study found that hUC-MSCs-Exos had a higher abundance of the above miRNAs, suggesting that hUC-MSCs-Exos inhibits chondrocyte inflammation may be through these miRNAs. Recently, miRNA-26a was reported to regulate cartilage ECM homeostasis, regulating chondrocyte proliferation and apoptosis by targeting genes related to cell cycle and cell survival [33, 34]. miRNA-26a can also inactivate NF-κB to inhibit the production of pro-inflammatory cytokines [35]. Excessive production of NO inhibits matrix synthesis and promotes the breakdown of cartilage and pain. Although various potential NO reduction approaches can be considered, selective inhibition of inducible nitric oxide synthase is preferred as a therapeutic strategy [36]. Rasheed et al. reported that miRNA-26a is a direct regulator of inducible nitric oxide synthase expression in chondrocytes, an important regulator of cartilage homeostasis, and a new target for therapy of OA [37]. miRNA-100-5p can bind to the 3'UTR region of mTOR, inhibit mTOR, promote autophagy, reduce the expression of inflammatory factors, maintain chondrocytes proliferation and increase ECM synthesis in OA chondrocytes [38]. Wu et al. found that miRNA-100-5p may be involved in the maintenance of articular cartilage through the mTOR autophagy signaling pathway, since the mTOR autophagy signaling pathway plays a key role in the progression of knee OA [39]. The miRNA-100-5p has at least 6 target genes, which are respectively IGF1R, FGFR3, PLK1, ID1, MMP13 and FLT1 [40]. The results of Gao ren's study showed that the level of miRNA-100-5p in plasma and synovial fluid of OA patients was lower than that of healthy controls, while the expression of MMP13 in articular cartilage was higher than that of controls, and MMP13 was the central node of the molecular network of cartilage degradation [38, 41]. The study of Sun et al. showed that let-7a binds to the 3'UTR of Chd4, which in turn promotes differentiation of mouse fibroblasts into chondrocytes [42]. In addition, functional enrichment analysis of miRNA-regulated genes can better identify miRNA-regulated pathways and involved biological processes [27].

In addition to autocrine interactions, the communication between chondrocytes and macrophages plays a crucial role in the onset and progression of OA through paracrine signaling. This signaling involves the secretion of inflammatory cytokines, tissue inhibitor of metalloproteinases, matrix metalloproteinases and growth factors, leading to inflammation of chondrocytes. Therapies targeting macrophages of synovial have been shown to protect against cartilage, synovitis damage and alleviate pain [21]. Chondrocytes were co-cultured with M1 macrophages utilizing permeable cell culture chambers. The cells can communicate through filaments that traverse the small pores of the membrane. This mechanism has been employed in research to explore intercellular signaling and potential clinical applications. Paracrine interactions between chondrocytes and macrophages are crucial in the pathogenesis of OA by secreting matrix metalloproteinases and inflammatory cytokines that ultimately result in cartilage degradation and destruction. The co-culture system was used to study the chondrocyte-macrophage interaction in this study. This co-culture model simulates the process of osteoarthritis well, which is closer to the occurrence of osteoarthritis *in vivo*. In our research, the addition of hUC-MSCs-Exos to M1 macrophages in the upper chamber showed down-regulation of the gene expression of *IL-1β* and *TNF-α*, up-regulation of *IL-10* and *ARG1*, and reversed the gene and protein expression of MMP13 and COL2A1of the chondrocytes seeded in the lower chamber. This is consistent with the function of bone marrow-derived MSCs-Exos reported by Zhang et al. [25]. Chondrocytes were co-cultured with pre-treated macrophages of mice, and it was observed that M2 type macrophages of mice increased the expression of chondrogenic genes, such as *COL2A1* and *sox9*. Moreover, M2 type macrophages of mice suppressed the expression of hypertrophic genes, such as *collagen X* and *runx2*. Recent research has attempted to elucidate the communication between macrophages and chondrocytes in OA progression. Samavedi et al. employed a comprehensive three-dimensional (3D) co-culture system and discovered that chondrocytes co-cultured with M1 macrophages secreted large amounts MMP1, MMP3, MMP9, MMP13, IL-6, IFN-γ, TNF-α, IL-8, and IL-1β [43]. It was also noted that in co-cultured macrophages, chondrocytes stimulated the expression of VEGF-A and IL-1β. Further investigation is required to determine the regulatory effect of macrophages through direct cell contact, as indirect co-culture method.

We mainly focused on the regulation of chondrocytes by miRNAs of hUC-MSCs-Exos. Therefore, we screened three types of genes: genes that were down-regulated when hUC-MSCs-Exos were added to IL-1β-induced osteoarthritis cartilage models; genes that were up-regulated when IL-1β was added to normal chondrocytes; genes related to osteoarthritis in the GeneCards database. We took the intersection of these three types of genes and screened out 22 key genes. KEGG pathway enrichment analysis was performed, and a total of 6 pathways were obtained (Figure 5E). Among them, PI3K-Akt and MAPK were important signaling pathways related to OA. PI3K/AKT involves 150 proteins and is a very complex and important signaling pathway [44]. The key downstream effectors, mTOR and NF-κB serve as master modulators responsible for initiation of autophagy and inflammation in OA [44, 45]. The PI3K/AKT/mTOR signaling mediated chondrocytes autophagy, inflammatory response, proliferation, and apoptosis affect pathophysiology of OA. Inhibitors of PI3K, AKT, and mTOR can attenuate the inflammation response and promote autophagy of chondrocytes in rats with OA [46]. PI3K/AKT/NF-κB is involved in many OA-associated events, such as survival of chondrocytes, chondrocyte inflammation, and chondrocyte catabolism. Studies have shown that IL-1β can stimulate the phosphorylation of PI3K and AKT [46, 47]. NF-κB promotes catabolic gene (MMP1, MMP9, and ADAMTS5) expression by binding to its promoter region and promotes the expression of destructive mediators and major pro-inflammatory of OA [48–51]. The MAPK can be activated by bacteria, physical stimulation, extracellular signals and inflammatory cytokines (such as IL-1β and IL-6) [52]. Upon activation, MAPK transduces extracellular signals to the nucleus and modulates the activity of transcription factors, ultimately leading to alterations in gene expression and induction of cellular responses [53]. The current body of research indicates a correlation between the MAPK signaling pathway and OA progression. Dysregulation of the MAPK signaling pathway can accelerate the inflammatory response, resulting in the release of numerous cartilage matrix-degrading enzymes and exacerbating cartilage degeneration [53]. Therefore, MAPK play an important role in the pathogenesis of OA [54–57].

## CONCLUSIONS

hUC-MSCs-Exos have anti-inflammatory effect on both IL-1β and co-incubation with macrophage induced human articular chondrocyte inflammation. hUC-MSCs-Exos may have the ability to resist chondrocyte inflammation in the treatment of OA.
